# Tobacco smoking predicts depression and poorer quality of life in heart disease

**DOI:** 10.1186/1471-2261-13-35

**Published:** 2013-05-24

**Authors:** Lesley Stafford, Michael Berk, Henry J Jackson

**Affiliations:** 1 Centre for Women’s Mental Health, Royal Women’s Hospital, Parkville, Australia; 2 Melbourne School of Psychological Sciences, University of Melbourne, Melbourne, Australia; 3 School of Medicine, Deakin University, Deakin, Australia; 4 ORYGEN Youth Health, Parkville, Australia; 5 Mental Health Research Institute, Parkville, Australia; 6 Department of Psychiatry, University of Melbourne, Melbourne, Australia

**Keywords:** Coronary artery disease, Depression, Smoking, Quality of life

## Abstract

**Background:**

We report on the prospective association between smoking and depression and health-related quality of life (HRQOL) in patients with coronary artery disease (CAD).

**Methods:**

Prospective study of 193 patients with assessment of depression occurring 3-, 6- and 9- months (T1, 2, and 3, respectively) following discharge from hospital for a cardiac event. HRQOL was assessed at T3. T1 depression was assessed by clinical interview; T2 and T3 depression was assessed by self-report. Smoking at time of cardiac event was assessed by self-report. Multivariate analyses controlled for known demographic, psychosocial and clinical correlates of depression.

**Results:**

Smoking at the time of index cardiac event increased the likelihood of being diagnosed with Major Depressive Disorder (MDD) at T1 by 4.30 [95% CI, 1.12-16.46; *p* < .05]. The likelihood of receiving a diagnosis of minor depression, dysthymia or MDD as a combined group was increased by 8.03 [95% CI, 2.35-27.46; *p* < .01]. Smoking did not reliably predict depression at T2 or T3 and did not reliably predict persistent depression. Smoking increased the likelihood of being classified as depressed according to study criteria at least once during the study period by 5.19 [95% CI, 1.51-17.82; *p* < .01]. Smoking independently predicted worse mental HRQOL.

**Conclusions:**

The findings support a role for smoking as an independent predictor of depression in CAD patients, particularly in the first 3 months post-cardiac event. The well-established imperative to encourage smoking cessation in these patients is augmented and the findings may add to the evidence for smoking cessation campaigns in the primary prevention of depression.

## Background

Tobacco smoking is a leading global cause of preventable morbidity and mortality [1]. Despite a steady recent decrease in smoking prevalence in the general population, smoking remains responsible for approximately 8% of the overall burden of disease and injury [2]. Although there is substantial recognition of the effect of smoking on physical morbidity, there is lesser appreciation of its connection to psychological morbidity, including depression. Smoking is disproportionately common among individuals with psychiatric disorders [3-6] and there is evidence that it is noxious to mental health [7-9]. Smoking also appears to influence some of the pathophysiological pathways that are germane to depression [10].

A reciprocal relationship exists between depression and smoking such that depression is more common in smokers than non-smokers, and smoking is more common in depressed versus non-depressed individuals. Studies in the general population have found that tobacco smoking is a risk factor for depression [11,12]. A recent study showed that the presence of smoking doubled the risk of development of a *de-novo* episode of major depression in women followed up for 10 years [13].

Both depression [14-16] and smoking [17,18] are risk factors for cardiac morbidity and mortality in patients with CAD; however, previous data on the association between tobacco smoking and depression in CAD patients are inconsistent. Some studies report no association [19-21] and others report that smoking is a risk factor for depression in CAD [22,23]. We conducted an analysis of data from a longitudinal study of depression in CAD to investigate the prospective association between smoking and depression. Based on the weight of evidence, we hypothesized that smoking would be associated with increased risk for the subsequent onset of depression among recently hospitalized CAD patients when known correlates of depression were controlled *a priori*. These correlates include clinical variables such as disease severity, body mass index (BMI), having undergone coronary artery bypass graft (CABG) surgery, diabetes, alcohol intake, functional disability, prior history of depression, Generalized Anxiety Disorder (GAD), neuroticism, and known socio-demographic factors such as gender, income, marital status and social support,). As a secondary outcome, we investigated the association between smoking and health-related quality of life (HRQOL). A deleterious impact of smoking on HRQOL has been reported in cross-sectional samples of the general population [24-26]. Only one prior longitudinal study has explicitly examined this association in CAD patients and showed that continued smoking after percutaneous transluminal coronary angioplasty (PTCA) significantly diminished the HRQOL benefits of this procedure [27]. We further hypothesized that smoking would be associated with worse HRQOL. The aims of the study were therefore to investigate the prospective association between smoking and depression and smoking and HRQOL in a cohort with cardiovascular disease.

## Methods

### Sample

Participants were recruited between May 2005 and March 2006 from a tertiary hospital in regional Australia that serves a catchment area shown to be representative of the broader Australian community [28]. All English-speaking, consenting patients who were hospitalized for PTCA, myocardial infarction (MI) or CABG during this time were eligible for participation. According to discharge diagnoses, 528 patients were treated for these presentations during the study period. Patients with different CAD presentations were regarded as a homogenous group based on evidence that the association between depression and CAD is found across all subgroups of CAD patients with similar prognostic effects for cardiac [14,29] and HRQOL [30,31] outcomes. Participants were recruited by postal invitation and follow-up phone-call 6 weeks post-discharge. Reasons for non-participation were death (*n* = 13), non-English speaking (*n* = 16), unable to consent (*n* = 9), medically unwell (*n* = 9), depression (*n* = 3) and no given reason (*n* = 249). Agreement to participate was obtained from 229 patients. The study received ethics approval from the Barwon Health Human Research and Ethics Committee and all participants gave written informed consent.

### Procedures

Data were collected at 3- (T1), 6- (T2) and 9 months (T3) after hospital discharge. Baseline assessment was undertaken 3 months post-discharge, rather than during admission, as well as to avoid potential confounding of the effects of acute illness and stress associated with hospitalization with the assessment of predictor variables and to minimize the burden on participants. Other studies [22,23,32] have highlighted difficulties in reliably identifying patients in whom depression will emerge, persist or worsen only on the basis of level of depressive symptomatology present during hospitalization. Structured clinical interviews were administered telephonically at T1. Telephonic administration of structured clinical interviews has been found to be valid and reliable [33,34]. For analyses, participants were assigned to a diagnostic group “major depressive disorder” (MDD) or to a broader group “any depressive disorder” (‘ADD’). The latter comprised, in addition to participants with MDD, participants with minor depression and dysthymia.

Participants were also mailed self-report questionnaires at T1, T2 and T3. T1 questionnaires assessed neuroticism, social support, and functional disability. The depression self-report measure was administered at T2 and T3. The structured clinical interview was used to establish a diagnosis while the self-report depression measure enabled tracking of depression change over time. HRQOL data were collected at T3. Relevant clinical and socio-demographic data were collected by self-report and from medical records at T1.

### Measures

Clinical data, including discharge diagnosis, antidepressant therapy at discharge, disease severity and the presence of hypertension and diabetes, were obtained from medical records. Disease severity was measured with left ventricular ejection fraction (LVEF). BMI was computed based on self-reported height and weight (kg/m^2^) at the time of the index event. Data on commencement of antidepressant therapy after index event, attendance at cardiac rehabilitation, marital status, annual household income, prior history of depression, and tobacco use and alcohol consumption at the index event were gathered by self-report. Alcohol consumption was considered excessive if greater than the quantity recommended by the National Heart Foundation of Australia (≤7 and 14 standard drinks per week for women and men, respectively) [35]. Low household income was categorized as ≤ $20 000 (Australian).

Clinical depression and GAD were assessed using the Mini International Neuropsychiatric Interview Version 5 (M.I.N.I.) [36,37], a diagnostic structured interview compatible with ICD-10 and DSM-IV criteria, and similar to the Structured Clinical Interview for DSM-IV Disorders (SCID) [38] in operation and principle. The M.I.N.I. has excellent psychometric properties and has been validated against the SCID-Patient Version [39] and the Composite International Diagnostic Interview (CIDI) [36,37,40,41]. M.I.N.I modules for depression, GAD and dysthymia were administered. Major depressive disorder (MDD) was diagnosed if participants fulfilled DSM-IV criteria of at least one core criterion (depressed mood or anhedonia) and at least four additional criteria within a 2-week duration. With the use of the same module, minor depression was diagnosed if participants fulfilled at least one core criterion and one to three additional criteria within a 2-week period.

Depressive symptomatology was measured with the 7-item depression subscale of the Hospital Anxiety and Depression Scale (HADS) [42]. The HADS has well-established psychometric properties [43,44] and is widely used in studies with cardiac patients [45-47]. Possible scores range from 0 to 21. Higher scores denote greater depressive symptomatology. Cronbach’s alpha at T1, T2 and T3 were .81, .82, and .84, respectively. In accordance with previous data [44,48-50], a score ≥ 8 was considered to indicate depression.

Social support was assessed with the psychometrically-robust [51-53] and widely-used [49,54-56] 12-item Multidimensional Scale of Perceived Social Support (MSPSS)[57]. This scale measures support from family, friends and a significant other. The total score ranges from 7 to 84 with higher scores indicating higher levels of support. Cronbach’s alpha was .93.

Neuroticism was measured with a 10-item scale from the self-report version of the NEO PI-R [58], the IPIP-NEO [59]. Scores range from 10 to 50 with higher scores indicating greater neuroticism. The IPIP-NEO has been shown to be reliable [60] and valid [58,61] and its factor structure has been confirmed [62,63]. Cronbach’s alpha was .88.

HRQOL was measured with the Short Form-36 (SF-36) [64], an inventory that assesses eight domains of HRQOL: physical functioning, bodily pain, fatigue, role limitations due to physical health problems, emotional functioning, role limitations due to emotional problems, social functioning and general health perception. Aggregate summary scores for physical functioning (PCS) and mental functioning (MCS) were calculated according to the standard algorithm [64]. Higher scores denote better functioning. Psychometric evidence suggests that the SF-36 is the most reliable, valid, and sensitive generic measure of HRQOL for use in CAD patients [65]. Cronbach’s alpha values for the eight subscales at T1 and T3 ranged from .80 to .92, and .81 to .93, respectively.

Functional disability was measured using the 10 items from the SF-36 that assess physical functioning. Items include activities such as walking one block and climbing a flight of stairs. In the original context these items assess limitations on physical activities. Here, the response format was altered to assess the severity of symptoms experienced when performing these physical tasks. The items are similar to measures like the Canadian Cardiovascular Society for angina severity [66] and the New York Heart Association classification criteria for the prescription of physical activity for cardiac patients [67]. Responses include *I do not experience symptoms*, *I experience mild symptoms*, *I experience severe symptoms*, and *I am not able to perform this activity*. Scores range from 0 to 30 with lower scores indicating greater functional disability.

### Analysis

Data were analysed using SPSS. All tests were two-tailed and *α* was .05. Univariate analyses comprised Chi-square and independent sample *t*-tests, as appropriate. Multivariate analyses to predict the association between smoking and depression used direct logistic regression. All predictor variables were entered in one block. Regression coefficients, Wald statistics, odds ratios (ORs) and 95% confidence intervals were calculated. Regression analyses for T1 comprised predicting MDD and ‘ADD’, as defined above. At T2 and T3, depression was defined by a HADS score ≥8. Two additional independent variables were created. Participants were assigned *a priori* to a ‘persistently depressed’ group if they were classified as depressed at T1, T2 and T3. Participants were assigned to an ‘ever depressed’ group if they were classified as depressed at T1, T2 or T3. For these two variables, membership of the ‘ADD’ group constituted T1 depression. Multivariate analyses to predict physical and mental HRQOL at T3 were performed using standard regression.

## Results

### Characteristics of the sample

T1 questionnaires were completed by 193 of the 229 recruited patients (84%; or 37% of all patients treated during the study period included those deceased and ineligible). According to discharge diagnoses, these 193 participants were treated for MI (n = 13, 7%), CABG (n = 91, 47%), PTCA (n = 35, 18%), MI and CABG (n = 21, 11%), and MI and PTCA (n = 35, 18%). [To facilitate analysis, this variable was categorized into CABG vs MI/PTCA]. Compared to non-participants, participants were significantly more likely to be male [*χ*^2^(1, *N* = 528) = 5.93, *P* = .02] and were, on average, younger [*t*(526) = 3.12, *P* < .01]. The mean (SD) age for participants versus non-participants was 64.14 (10.37) versus 67.31 (11.70), respectively. Twenty-eight participants did not return their questionnaires for an unspecified reason, 3 withdrew due to physical illness and 4 withdrew due to depression. There were no significant gender differences between those who completed T1 questionnaires and those who did not [*χ*^2^(1, *N* = 229) = .64, *P* = .42], but non-respondents were, on average, significantly younger than respondents [*t*(227) = −2.13, *P* < .03]. The mean (SD) age for non-responders was 59.94 (13.22). At T2, 189 of the 193 T1 participants remained. By T3, 184 of the 193 T1 participants remained (95%; or 35% of the original patient group (184 of 528)). One participant had formally withdrawn due to depressive illness and 8 participants did not return their questionnaires for unspecified reasons. There were no gender or age differences between participants who completed T2 and T3 questionnaires and those who did not. Baseline clinical, socio-demographic, and psychosocial data for the sample and for non-smokers versus smokers are described in Table 1.

**Table 1:** Characteristics of sample at T1

	**Non-smoker (*n*** **= 166)**	**Smoker (*n*** **= 27)**	**Total (*N*** **= 193)**
**Male,*n*(%)**	137 (83)	19 (70)	156 (81)
Age (years), M±SD (range)***	65.23±9.81	57.44±11.40	64.14±10.37 (38–91)
**Income < AU$20 000,*n*(%)**	54 (33)	9 (33)	63 (33)
**Married,*n*(%)**	131 (79)	15 (56)	146 (76)
**Hypertension,*n*(%)**	127 (77)	23 (85)	150 (78)
**Diabetes,*n*(%)**	40 (24)	5 (19)	45 (23)
CABG,*n*(%)**	104 (63)	8 (30)	112 (58)
**BMI (kg/m**^**2**^**), M±SD (range)**	28.07±4.28	27.55±3.92	28.00±4.22 (20–40)
**LVEF < 30%*n*(%)**	11 (7)	0 (0)	11 (8)
**Alcohol - Excessive**	34 (12)	6 (22)	40 (21)
**Functional status, M±SD (range)**	24.81±4.57	23.97±6.33	24.09±6.11 (0–30)
MDD***	23 (14)	12 (44)	35 (18)
ADD***	36 (33)	18 (67)	54 (28)
GAD*	27 (16)	10 (37)	37 (19)
**History of depression**	57 (34)	10 (37)	67 (35)
**Antidpressants at T1,*n*(%)**	17 (10)	3 (11)	20 (11)
**Antidepressants T1-T3,*n*(%)**	13 (8)	3 (11)	16 (8)
Neuroticism, M±SD (range)**	26.48±8.50	32.44±10.56	27.32±9.02 (10–50)
Social support, M±SD (range)**	70.25±11.19	63.89±12.34	69.36±11.54 (21–84)
**Attended cardiac rehabilitation**^**†**^,*n*(%)*	121 (76)	13 (50)	134 (72)

*ADD* = Any depressive disorder; *BMI* = body mass index; *CABG* = coronary artery bypass graft surgery; *GAD* = Generalized Anxiety Disorder; *LVEF* = left ventricular ejection fraction; *MDD* = Major Depressive Disorder.

^**†**^**Known for n = 187.**

*P < .05 **P < .01 *** *P* < .001.

### Association between smoking and depression

Twenty-seven participants (14%) were smokers at the time of index event. At T1, 35 (18%) participants were diagnosed with MDD and 54 (28%) with ‘ADD’. Univariate analyses (Table 1) showed that compared to non-smokers, participants who were smokers at the time of the index cardiac event were significantly younger [*t*(191) = 3.73, *P* < .001]; less likely to have undergone CABG [*χ*^2^(1, *N* = 193) = 10.4, *P* = .001], less likely to attend cardiac rehabilitation [*χ*^2^(1, *N* = 187) = 6.98, *P* = .02], perceived less social support [*t*(191) = 2.70, *P* < .01], reported higher levels of neuroticism [*t*(31.72) = −2.79, *P* < .01] and were more likely to receive a diagnosis of GAD [*χ*^2^(1, *N* = 193) = 6.47, *P* = .01], ‘ADD’ [*χ*^2^(1, *N* = 193) = 23.32, *P <* .001] or MDD [*χ*^2^(1, *N* = 193) = 14.64, *P* < .001] at T1 (i.e., 3 months later).

Logistic regression analyses to predict depression are shown in Table 2 (MDD, ‘ADD’, and T2 Depression) and Table 3 (T3 Depression, ‘Persistently depressed’, and ‘Ever depressed’). Inspection of the ORs showed that smoking at the time of index cardiac event increased the likelihood of being diagnosed with MDD at T1 by 4.30 [95%CI, 1.12-16.46; *P* < .05], an OR second only to that associated with a diagnosis of GAD. In the analysis to predict ‘ADD’ at T1, smoking had an OR of 8.03 [95% CI, 2.35-27.46; *P* < .01] and was the strongest independent predictor of outcome of all the available variables. At T2 and T3, 24 (13%) and 29 (16%) participants, respectively, reported a HADS score ≥ 8. Smoking did not reliably predict a depression at T2 and T3. Fourteen participants (8%) were assigned to the ‘persistently depressed’ group and 60 (33%) were assigned to the ‘ever depressed’ group. Smoking was not reliably associated with classification as ‘persistently depressed’. Smoking increased the likelihood of being classified as ‘ever depressed’ by an OR of 5.19 [95% CI, 1.51-17.82; *P* < .01] and was again the most important predictor of depression in this analysis.

**Table 2:** Direct logistic regression analyses to predict the association between smoking and depression (MDD, ’ADD’ and T2 Depression)

	**T1 MDD (*N*** **= 193)**						**T1 ‘ADD’ (*N*** **= 193)**						**T2 Depression (*N*** **= 189)**					
	**B**	**SE**	**Wald**	**OR**	**95% CI**						**95% CI**						**95% CI**	
**Variable**					**Lower**	**Upper**	**B**	**SE**	**Wald**	**OR**	**Lower**	**Lower**	**B**	**SE**	**Wald**	**OR**	**Lower**	**Upper**
**Male**	-.24	.64	.14	.79	.23	2.77	.19	.59	.10	1.21	.38	3.83	.57	.70	.65	1.76	.44	6.97
**Married**	.79	.74	1.15	2.21	.52	9.43	.48	.63	.596	1.62	.47	5.56	.67	.74	.81	1.96	.46	8.39
**Income**	.57	.61	.86	1.76	.53	5.82	.12	.54	.05	1.12	.39	3.24	-.20	.65	.09	.82	.23	2.96
**LVEF**	−18.86	1.10	.00	.00	.00	.	-.42	1.22	.12	.65	.06	7.12	−18.51	1.13	.00	.00	.00	.
**BMI**	-.04	.06	.41	.96	.85	1.09	.04	.05	.74	1.05	.95	1.15	-.02	.07	1.00	.98	.85	1.12
**Diabetes**	-.35	.66	.29	.70	.19	2.54	-.51	.58	.80	.60	.19	1.85	-.65	.71	.84	.52	.13	2.09
***Smoking***	***1.46***	***.69***	4.52*	***4.30***	***1.12***	***16.46***	***2.08***	***.63***	11.05**	***8.03***	***2.35***	***27.46***	***.16***	***.71***	***.05***	***1.17***	***.29***	***4.69***
**Alcohol - excessive**	-.70	.66	1.13	.50	.14	1.81	-.07	.55	.02	.93	.32	2.71	.05	.64	.01	1.05	.30	3.71
**History of depression**	.94	.53	3.13	2.56	.90	7.23	.75	.46	2.65	2.12	.86	5.22	.68	.56	1.51	1.98	.67	5.88
**GAD**	1.87	.60	9.56**	6.50	1.98	21.09	1.81	.55	10.74**	6.09	2.07	17.93	.05	.69	.01	1.06	.27	4.11
**Neuroticism**	.08	.04	4.37*	1.08	1.01	1.16	.09	.03	7.67**	1.09	1.03	1.16	.08	.04	4.32*	1.09	1.01	1.17
**Social support**	.01	.03	.15	1.01	.96	1.06	.01	.02	.20	1.01	.97	1.05	-.06	.02	6.35*	.94	.90	.99
**Functional status**	-.07	.04	2.64	.93	.86	1.01	-.08	.04	5.15*	.92	.86	.99	-.11	.05	5.76*	.90	.82	.98
**CABG**	.91	.53	2.94	2.49	.88	7.04	1.10	.45	6.07*	3.01	1.25	7.24	1.23	.57	4.64*	3.43	1.12	10.54
**Constant**	−3.83	3.25	1.38	.02			−5.43	2.69	4.06	.00			.96	3.06	1.00	2.62		

*ADD* = Any depressive disorder; *BMI*  body mass index; *CABG* = Coronary artery bypass graft surgery; *GAD* = Generalized Anxiety Disorder; *Income* = Annual household income ≤ AU$20 000; *LVEF* = left ventricular ejection fraction <30%; *MDD* = Major Depressive Disorder.

*P < .05 **P < .01 *** *P* < .001.

**Table 3:** Direct logistic regression analyses to predict the association between smoking and depression (T3 Depression, ‘Persistently depressed’ and ‘Ever depressed’)

	**T3 Depression (*N*** **= 184)**						**‘Persistently depressed’ (*N*** **= 184)**						**‘Ever depressed’ (*N*** **= 184)**					
	**B**	**SE**	**Wald**	**OR**	**95% CI**						**95% CI**						**95% CI**	
**Variable**					**Lower**	**Upper**	**B**	**SE**	**Wald**	**OR**	**Lower**	**Lower**	**B**	**SE**	**Wald**	**OR**	**Lower**	**Upper**
**Male**	.31	.68	.21	1.37	.360	5.20	.04	.82	.00	1.04	.21	5.15	.82	.62	1.72	2.26	.67	7.66
**Married**	1.87	.93	4.11*	6.52	1.06	39.89	.60	1.10	.31	1.82	.235	14.63	.91	.68	1.79	2.48	.66	9.39
**Income**	-.81	.73	1.23	.45	.11	1.86	.01	.81	.00	1.01	.21	4.94	.01	.55	.00	1.01	.35	2.96
**LVEF**	−18.54	1.10	.00	.00	.00	.	−17.66	1.13	.00	.00	.00	.	−1.08	1.19	.84	.34	.03	3.45
**BMI**	.10	.06	3.04	1.11	.99	1.25	-.05	.09	.33	.95	.80	1.13	.03	.05	.47	1.03	.94	1.14
**Diabetes**	1.11	.63	3.12	3.05	.88	10.51	.15	.84	.03	1.16	.23	5.99	-.78	.58	1.83	.46	.15	1.42
***Smoking***	***.43***	***.74***	***.34***	***1.54***	***.36***	***6.58***	***.06***	***.91***	***.01***	***1.07***	***.18***	***6.32***	***1.65***	***.63***	6.84**	***5.19***	***1.51***	***17.82***
**Alcohol - excessive**	.18	.72	.06	1.19	.29	4.91	-.61	.93	.43	.54	.09	3.35	.34	.56	.36	1.40	.47	4.23
**History of depression**	.62	.55	1.27	1.86	.63	5.55	1.69	.77	4.87*	5.41	1.21	24.23	.15	.47	.10	1.16	.46	2.93
**GAD**	1.38	.65	4.53*	3.99	1.12	14.28	1.04	.876	1.45	2.83	.52	15.48	1.43	.56	6.45*	4.16	1.38	12.48
**Neuroticism**	.06	.04	2.59	1.06	.99	1.14	.04	.05	.52	1.04	.94	1.14	.13	.03	14.41***	1.14	1.06	1.21
**Social support**	-.07	.03	5.91*	.94	.89	.99	-.04	.03	1.77	.96	.91	1.02	-.02	.02	.62	.98	.94	1.03
**Functional status**	-.13	.05	5.96*	.88	.80	.98	-.06	.06	.95	.95	.84	1.058	-.18	.05	15.52***	.84	.77	.92
**CABG**	.49	.56	.78	1.63	.55	4.84	1.13	.72	2.51	3.11	.76	12.653	.75	.44	2.88	2.11	.89	4.98
**Constant**	−2.01	3.07	.43	.13			.04	.82	.00	1.04	.21	5.153	−2.15	2.56	.71	.12		

*ADD* = Any depressive disorder; *BMI* = body mass index; *CABG* = Coronary artery bypass graft surgery; *GAD* = Generalized Anxiety Disorder; *Income* = Annual household income ≤ AU$20 000; *LVEF* = left ventricular ejection fraction <30%; *MDD* = Major Depressive Disorder.

*P < .05 **P < .01 *** *P* < .001.

### Association between smoking and HRQOL

Correlations between T1 demographic, psychosocial and clinical variables and T3 mental and physical HRQOL are shown in Table 4. Multivariate analyses to investigate the association between smoking and HRQOL at T3 are shown in Table 5. The model to predict MCS explained 47% of the variance in outcome [*F*(14,184) = 10.85, *P* < .001] and showed that smoking was significantly associated with MCS (*P* < .001), explaining approximately 4.8% of unique variance. Neuroticism was the most important predictor and contributed approximately 6.7% of unique variance. The model to predict PCS explained 46% of the variance in outcome [*F*(14,184) = 10.24, *P* < .001]. Smoking was not an independent predictor of PCS (*P* = .98).

**Table 4:** Correlations between T1 demographic, psychosocial and clinical variables and T3 mental and physical HRQOL

**Variable**	**1**	**2**	**3**	**4**	**5**	**6**	**7**	**8**	**9**	**10**	**11**	**12**	**13**	**14**	**15**	**16**
**1. Male**	1															
**2. Married**	.28***	1														
**3. Income**	-.14	-.27***	1													
**4. LVEF**	.06	-.02	.07	1												
**5. BMI**	-.06	.09	-.01	-.05	1											
**6. Diabetes**	-.04	-.06	.17*	.02	.10	1										
***7. Smoking***	*-.11*	-.19***	*.01*	*-.10*	*-.04*	*-.05*	*1*									
**8. Alcohol -excessive**	.18*	.08	-.06	-.07	-.07	-.13	.02	1								
**9. History of** **depression**	-.06	-.09	.05	-.09	.09	-.04	.02	.00	1							
**10. GAD**	-.03	.00	.05	-.12	.04	-.08	.18*	.01	.23***	1						
**11. Neuroticism**	-.10	.03	.15*	-.11	.04	.03	.23***	.00	.34***	.49**	1					
**12. Social support**	.06	.34***	-.10	.04	.00	.04	-.20***	.05	-.08	-.19**	-.12	1				
**13. Functional status**	.20***	.19***	-.42***	-.07	-.07	-.20***	.02	.11	-.16*	-.06	-.17*	.11	1			
**14. CABG**	-.12	-.06	0.08	-.03	.00	-.10	.23**	.06	-.03	.09	.13	.00	.01	1	-.15*	
**15. MCS**	.100	.02	-.06	.09	-.26***	-.08	-.36***	.07	-.32***	-.41***	-.54***	.22***	.21***		1	
**16. PCS**	.16*	.16*	-.29***	-.01	-.07	-.23***	-.04	.06	-.28***	.00	-.21***	.06	.61***	.18***		1

*BMI* = body mass index; *CABG* = Coronary artery bypass graft surgery; *GAD* = Generalized Anxiety Disorder; *Income* = Annual household income ≤ AU$20 000; *LVEF* = left ventricular ejection fraction <30%; *MCS* = Mental Component Summary score of the SF-36; *PCS* = Physical Component Summary score of the SF-36.

*P < .05 ** *P* < .01 *** *P* < .001.

**Table 5:** Multiple regression analyses to predict the association between smoking and HRQOL at T3

	**Physical HRQOL at T3 (*N*** **= 184)**					**Mental HRQOL at T3 (*N*** **= 184)**				
**Variable**				**95% CI for β**					**95% CI for β**	
	**B**	**SE**	**β**	**Lower**	**Upper**	**B**	**SE**	**β**	**Lower**	**Upper**
**Constant**	2191.21	471.57		1260.29	3122.14	5094.85	530.41		4047.77	6141.92
**Male**	−1.29	134.42	.00	−266.64	264.06	−78.07	151.19	-.03	−376.53	220.39
**Married**	82.47	135.80	.04	−185.61	350.56	−77.48	152.75	-.03	−379.01	224.06
**Income**	−3.39	120.15	-.00	−240.57	233.80	151.72	135.14	.07	−115.06	418.50
**LVEF**	67.40	212.32	.02	−351.75	486.55	−47.46	238.81	-.01	−518.90	423.99
**BMI**	2.54	11.90	.01	−20.95	26.02	−50.37	13.38	-.22***	−76.78	−23.95
**Diabetes**	−240.51	120.41	-.12*	−478.22	−2.81	−148.65	135.44	-.07	−416.01	118.72
***Smoking***	***−3.08***	***154.25***	***-.00***	***−307.58***	***301.42***	***−687.49***	***173.49***	-.24***	***−1029.98***	***−345.01***
**Alcohol - excessive**	−15.11	127.08	-.01	−265.97	235.75	125.54	142.93	.05	−156.62	407.69
**History of depression**	−325.52	114.85	-.18**	−552.24	−98.79	−252.56	129.18	-.12	−507.57	2.45
**GAD**	249.36	146.24	.11	−39.33	538.04	−378.52	164.48	-.15*	−703.22	−53.82
**Neuroticism**	−10.02	6.79	-.10	−23.42	3.38	−35.86	7.64	-.33***	−50.93	−20.79
**Social support**	-.02	.04	-.03	-.09	.054	.06	.04	.09	-.02	.15
**Functional status**	.06	.01	.55***	.05	.074	.02	.01	.12	-.00	.03
**CABG**	−248.66	104.18	-.14	−454.33	−43.00	−106.99	117.18	-.05	−338.31	124.33

*BMI* = body mass index; *CABG* = Coronary artery bypass graft surgery; *GAD* = Generalized Anxiety Disorder; *HRQOL* = Health related quality of life; *Income* = Annual household income ≤ AU$20 000; *LVEF* = left ventricular ejection fraction <30%.

*P < .05 ** *P* < .01 *** *P* < .001.

## Discussion

Our findings indicate that in the presence of well-known confounders, exposure to smoking is independently associated with a 4.30- and a 8.03-fold increase in odds for MDD and a diagnosis of minor depression, dysthymia or MDD as a combined group (‘ADD’), respectively, 3 months following cardiac hospitalization. Smoking increased the likelihood of being classified as depressed according to study criteria at least once during the 9 months of the study period by an OR of 5.19. Despite this finding, there was no significant association between smoking and depression at T2 or T3, and given that smoking status and depression were measured simultaneously at T1, an independent prospective association cannot be clearly concluded from these data. This study provides evidence consistent with the hypothesis that tobacco smoking is an independent predictor of depression and adds to the evidence that smoking is noxious to mental health [68]. Consistent with prediction, smoking was associated with worse mental HRQOL. There was, however, no association between smoking and physical HRQOL. These findings are similar to Rumsfeld et al. (2003) [69] who reported that tobacco smoking was independently predictive of 6-month mental but not physical HRQOL in CAD patients who underwent revascularization.

The mechanisms through which smoking is associated with depression are likely to be complex, involving bio-genetic, psychological and environmental factors. Indeed, studies in depression have supported a tri-directional relationship driven by mutually reinforcing effects and shared causal factors [70]. These factors include genetic and neural connectivity variables that are common to both depression and smoking; smoking-induced neurobiological changes that might predispose to depression; the transient alleviation of depressive symptoms and psychotropic side effects with smoking; and increased smoking as part of an agitated mental state.

Homeostatic compensation ensures that over time and with adaptation, agents that initially induce short-term alterations in mood later tend to produce contrary effects. For example, the acute effects of alcohol (i.e., anxiolysis and sleep induction) are characteristically the converse of the withdrawal pattern (i.e., anxiety and insomnia). Cigarette smoking can be conceptualised as a chronic dysphoric withdrawal state punctuated by brief reinforcing intoxications. Smoking addiction, in common with other addictions, is mediated via the dopaminergic reward pathway, and dopamine has a key role in depression [71]. Smoking dysregulates the striatal D2 receptor, which may play a shared role in both its pathway to addiction and its effect on mood [72].

The status of both tobacco smoking and depression as risk factors for cardiac morbidity and mortality is supported by evidence of shared pathways. For instance, cigarette smoke contains high amounts of oxidative free radicals, including nitric oxide [73]. These free radicals have a complex effect on the oxidative defence system, inducing a direct oxidative attack and simultaneously upregulating antioxidant defences [73]. Oxidative stress is implicated in the pathophysiology of both depression [74] and cardiovascular disease [75]. Cigarette smoking has also been associated with raised levels of C-reactive protein [76], suggesting the stimulation of a chronic inflammatory state, which again, is described in both depression and cardiovascular disorders [77,78].

Strengths of the study include its prospective design, the low attrition rate and the inclusion of well-known confounders of depression such as history of depression, neuroticism, anxiety and functional status. We acknowledge that the generalizability of our findings is limited by sample bias. Since respondents were more likely to be male and were on average younger than non-respondents, these findings should be cautiously applied to older, female CAD patients. Indeed, there is evidence that women and men with CAD have different medical, functional and psychosocial profiles [80–82]. Also, taking into account all patients treated during the study period, regardless of eligibility for the study, the compliance rate was low (193 of 528 = 37%). It is possible that patients with depression were less likely to participate in the study potentially resulting in an underestimation of the effect of smoking. Duration and exposure of smoking prior to depression onset are unknown. Baseline measures were not conducted prior to the cardiac interventions. Exact depression status was not known at the time of index cardiac event for the reasons explained earlier, but prior history of depression, the most important risk factor for subsequent depression, was included in all multivariate analyses. Still, it is plausible that depression led to tobacco use and that smokers had more depressive symptoms before their cardiac events. It is noted that LVEF can be an imprecise measure of disease severity but was the most consistently available measure in the medical records. We acknowledge that although ORs were significant and notable, the confidence intervals were wide. This has implications of imprecision in estimating the true effect size and may be addressed in future studies with larger sample sizes.

Individuals often need risk to be personalized before a health warning is internalized to the point that it precipitates a decision to act. The message that smoking is an independent predictor of depression in someone who experiences the distress of depression, may be a catalyst for change. This information may work in parallel with the notion that the diagnosis of a smoking-related physical illness can precipitate motivation for smoking discontinuation. The demonstration of the association between smoking and mood and HRQOL augments the well-established physical imperative to encourage smoking cessation in patients with CAD [79]. This message similarly needs to be routinely communicated to depressed individuals.

## Conclusions

Adjusted for well-known confounders of depression, smoking was independently associated with depression in patients with heart disease. Smoking was an independently associated with poor mental HRQOL. These data reinforce the imperative to encourage smoking cessation in patients with heart disease, and add to the evidence for smoking cessation campaigns in the primary prevention of depression [8].

## Abbreviations

ADD: Any depressive disorder; BMI: Body mass index; CABG: Coronary artery bypass graft surgery; CAD: Coronary artery disease; CIDI: Composite International Diagnostic Interview; DSM-IV: Diagnostic and Statistical Manual Version 4; GAD: Generalized Anxiety Disorder; HADS: Hospital Anxiety Depression Scale; HRQOL: Health related quality of life; LVEF: Left ventricular ejection fraction; MCS: Mental functioning aggregate summary score of the SF-36; MI: Myocardial infarction; MDD: Major depressive disorder; MSPSS: Multidimensional Scale of Perceived Social Support; MINI: Mini International Neuropsychiatric Interview Version 5; PCS: Physical functioning aggregate summary score of the SF-36; PTCA: Percutaneous transluminal coronary angioplasty; SCID: Structured Clinical Interview for DSM-IV; SF-36: Short Form-36.

## Competing interests

The authors declare that they have no competing interests.

## Authors’ contributions

LS collected and analysed the data. LS, MB, HJ and LS participated in the conception and design of the study. LS wrote the initial draft of the manuscript, MB and HJ participated in further manuscript writing. All authors read and approved the final manuscript.

## Pre-publication history

The pre-publication history for this paper can be accessed here:

http://www.biomedcentral.com/1471-2261/13/35/prepub
